# Fluorescent Yellow Urine Induced by Vonoprazan Fumarate: A Case Report of a Benign Phenomenon

**DOI:** 10.7759/cureus.101472

**Published:** 2026-01-13

**Authors:** Lei Sheng, Xuening Li

**Affiliations:** 1 Department of Clinical Pharmacology, Zhongshan Hospital, Fudan University, Shanghai, CHN

**Keywords:** clinical trial, drug-induced effect, urine discoloration, urine fluorescence, vonoprazan fumarate

## Abstract

An abnormal color in urine may be caused by various factors, including foods with high levels of vitamins, certain medications that metabolize in the body to produce fluorescent substances, or certain metabolic disorders. Although abnormal urine, such as black, green, pink, or purple urine, is commonly reported, fluorescent urine is rare and typically observed under ultraviolet light. This case reports a healthy participant with persistent fluorescent yellow urine after receiving vonoprazan fumarate (TAK-438) under visible lights. A 25-year-old healthy female participated in a Phase I clinical trial of vonoprazan fumarate. After the oral dose of vonoprazan fumarate, her urine showed intensely fluorescent yellow in urine collection intervals under visible light. During the whole study, she had a standard diet and did not experience any adverse event. Her urinalysis showed color in yellow or mild yellow, normal pH and density, normal urobilinogen, and absence of bilirubin. This fluorescent phenomenon resolved spontaneously after drug washout. This case presents a benign, drug-induced effect in urine color, which is likely caused by vonoprazan fumarate and its metabolites. It is essential for clinicians to be aware of this effect to avoid unnecessary invasive diagnostic procedures or patient anxiety.

## Introduction

Vonoprazan fumarate, a novel potassium-competitive acid blocker (P-CAB), was first approved in Japan in December 2014 and has been widely used for suppressing gastric acid secretion by competitively inhibiting the potassium-binding site of H+, K+-ATPase [1]. While its safety, pharmacokinetic, and pharmacodynamic profiles have been well-characterized in previous studies [2], the unexpected visual abnormality in urine color remains unreported. Under normal physiological conditions, urine color is primarily determined by urochrome and urobilin; however, deviations from this norm can be a sign of an underlying pathological condition, such as an infectious disease, a metabolic disorder, or the presence of exogenous substances [3].

Among these deviations, urine fluorescence that is typically observed under ultraviolet (UV) light is a particularly alarming sign, which immediately prompts clinical concern. It is historically linked to severe toxicological emergencies, such as ethylene glycol poisoning [4,5], or complex metabolic disorders like porphyrias [6,7]. In this article, we present a case of persistent fluorescent yellow urine observed in a healthy volunteer who participated in a Phase I clinical trial of vonoprazan fumarate, aiming to highlight the importance of differentiating a benign pharmacologic phenomenon from a pathological etiology.

## Case presentation

**Figure 1:**
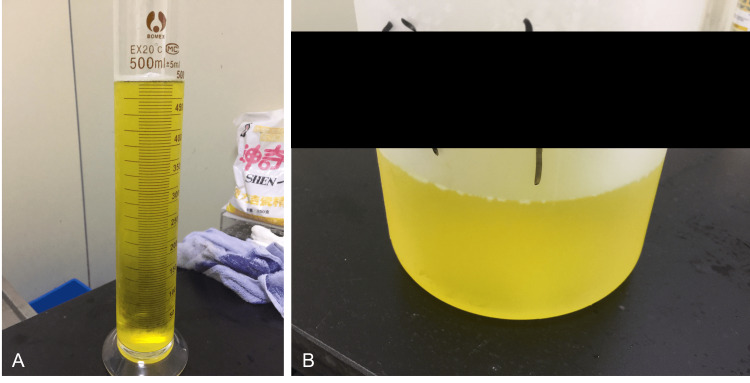
Urine from a 25-year-old woman after oral administration of vonoprazan fumarate (TAK-438). A persistent yellow fluorescence in urine is shown under visible light in a graduated cylinder (A) and a urine barrel (B).

A 25-year-old healthy female participated in a Phase I clinical trial of vonoprazan fumarate. She was admitted to the Phase I ward on day 1 and received a single dose of 20 mg vonoprazan fumarate on day 1 (Table 1). Following a pharmacokinetic blood sampling period of 48 hours that continued until the morning of day 3, she received twice-daily doses on days 3-9 (Table 1). Her urine showed a persistent and intensely fluorescent yellow color after dosing under visible light, both in a graduated cylinder (Figure 1A) and a urine barrel (Figure 1B). This fluorescence was readily apparent to the naked eye without ultraviolet light.

**Table 1:** Timeline of study procedures, dosing, and pharmacokinetic (PK) sampling

Period	Visit	Activity / dosing
Screening	Day -28 to -2	Screening for eligibility
Check-in	Day -1	Enrollment and initiation of the standard diet
Treatment	Day 1	Initial single 20 mg oral dose; PK sampling (blood and urine)
	Days 2 to 3	Continuation of 24–48-hour PK sampling
	Days 3 to 8	Multiple-dose regimen (20 mg BID)
	Day 9	Last single dosing and PK sampling; laboratory tests
	Days 10 to 11	Continuation of PK sampling
Check-out	Day 11	Final laboratory tests; ward departure
Follow-up	Day 18	Telephone follow-up to assess long-term safety

Her urine outputs were within normal range. Her laboratory tests (hematology, blood biochemistry, coagulation function, and urinalysis) on day 3, day 9, and day 11 showed normal results or abnormalities without clinical significance. Urinalysis showed color in yellow or mild yellow, normal pH and density, normal urobilinogen, and absence of bilirubin. She had a standard diet from day -1 (check-in) through to day 11 (check-out). She did not report discomfort or adverse symptoms in either treatment period or follow-up period and did not take any other medications besides vonoprazan fumarate.

## Discussion

Urine fluorescence is a distinct clinical finding that necessitates a careful differential diagnosis to distinguish between benign dietary or pharmacological causes and life-threatening conditions. Typically, mild yellow fluorescence may result from excessive intake of certain vitamins, notably high levels of riboflavin (vitamin B2), which naturally exhibits intrinsic fluorescent properties [8]. Beyond vitamin intake, various medications such as rifampin (red/orange), methylene blue (blue-green), and nitrofurantoin (brown to black) are well-documented to induce benign urine discoloration that may lead to clinical alarm if unrecognized [3,8]. Recognizing these non-pathological variations is essential for differential diagnosis, and the fluorescent yellow urine observed with vonoprazan fumarate adds a unique visual manifestation to this spectrum of drug-induced effects. By contrast, persistent urine fluorescence is a rare clinical phenomenon that may indicate serious intoxication, such as ethylene glycol poisoning. In such cases, the fluorescence is attributable to sodium fluorescein, an additive in antifreeze formulations designed to facilitate detection under UV light (i.e., Wood’s lamp) [4,5]. A similar fluorescence is also observed in metabolic disorders like porphyria, which is characterized by the excretion of porphyrins that fluoresce upon UV exposure [6,7].

In the present case, the persistent yellow fluorescence was observed under standard ambient lighting, distinct from the UV-dependent fluorescence typical of ethylene glycol poisoning or porphyrins. During the study, the participant had a standard diet and took no concomitant medications, effectively ruling out the possibility of hypervitaminosis or other drug-induced causes. Causality was assessed using the Adverse Drug Reaction Probability Scale (Naranjo), obtaining a total score of 8 based on the temporal onset after dosing, resolution after drug washout, a positive re-challenge after the multiple dose, exclusion of dietary or concomitant medication factors, and objective visual confirmation. This result categorizes the fluorescent yellow urine as a “probable” adverse reaction to vonoprazan fumarate. Consequently, these findings strongly support a benign cause associated with the administration of vonoprazan fumarate. Structurally, vonoprazan fumarate is a pyrrole derivative containing a benzene ring and unsaturated bonds [1]. These structural components, particularly the conjugated aromatic systems, are hypothesized to absorb light and re-emit it as fluorescence, although this remains unproven without specific spectroscopic analysis of vonoprazan fumarate and its metabolites. As vonoprazan fumarate is extensively metabolized and excreted [2], the fluorescence is likely due to the parent drug or its metabolites that possess fluorescent properties. Potential contributing factors for the intensity of this fluorescent phenomenon may include participant-specific variables, such as variations in drug metabolism (e.g., genetic polymorphisms of CYP2C19 and CYP3A4) and renal excretion rates, or the presence of underlying metabolic disorders. Identifying this benign effect is essential to prevent confusion with pathological signs of intoxication or metabolic disorders. 

However, such a dramatic visual presentation could be mistaken for serious etiologies by unaware clinicians. Misinterpretation could lead to unnecessary anxiety for the patient and costly, invasive diagnostic procedures.

## Conclusions

This case provides an important and unusual example of a benign, drug-induced phenomenon in urine color. Such a dramatic visual presentation could be mistaken for more serious etiologies, leading to an unnecessary and invasive diagnostic procedure; thus, clinicians should be aware of benign pharmacologic effects that can mimic pathological signs. Recognizing this harmless effect of vonoprazan fumarate is crucial to prevent unnecessary patient anxiety and avoid costly investigations.
